# Indiana State Dental Association

**Published:** 1861-07

**Authors:** J. B. Smith


### Proceedings of Societies.

---

### INDIANA STATE DENTAL ASSOCIATION.

INDIANAPOLIS, Jan. 2d, 1861.

Indiana State Dental Association met in the room of the Young Men's Christian Association, at 2 o'clock, P. M., 1st Vice-President, Dr. P. G. C. Hunt, in the chair.

Dr. Jno. F. Johnston, of Indianapolis, announced the death of Dr. C. A. Harris, of Baltimore, and Dr. John Hood, of Greensburg, late a member of this society—whereupon, Drs. Johnston, J. F. Canine, and P. W. Morris were appointed a committee to report resolutions expressive of the sentiments of this body in relation thereto.

The minutes of the last session were read.

The Committee on Order of Business reported:

I. Election of new members.

II. Reports of Committees.

III. Reading of essays, and remarks on the same.

IV. Discussions on the following subjects, in regular order:

1. Treatment of the fangs of teeth, preparatory to filling.
2. Preparation of the gold and the best mode of introducing it into the fang.
3. Filling ordinary cavities.
4. Mechanical Dentistry in all its various branches.

V. Miscellaneous Business.

VI. Election of Officers for ensuing term.

The report was adopted.

The Secretary made his annual report, which was concurred in.

The Treasurer made his report, which was referred to the Auditing Committee.

Dr. W. H. Atkinson, of Cleveland, Ohio, who was present by special invitation, was, on motion, elected to honorary membership. The Doctor responded his thanks in a laconic and interesting speech.

A colloquial and interesting discussion was then had upon the different topics, eliciting much valuable practical information, though few new ideas of importance.

On account of the small attendance of members, owing to a misunderstanding as to the time of meeting, much important business was passed over.

At the request of the association, Dr. Atkinson read an essay on the subject of labor. The thanks of the association were voted to Dr. A., and a copy of the essay asked and obtained for the archives and for publication.

A letter from Dr. Jno. T. Toland, of Cincinnati, was read, also, a despatch from the President, Dr. J. Knapp, of Fort Wayne, regretting their necessary absence from the meeting.

The following reports were read and unanimously adopted:

"WHEREAS, the death of Prof. Chapin A. Harris, of Baltimore, has made the heart of every member of this association, and, as we believe, the entire dental profession, feel sad; and, whereas, we have always regarded Dr. Harris as the great leading spirit in placing our profession in its present honorable position—his works being text-books for us all—we with sorrowful hearts desire, while paying this last sad tribute of respect, to acknowledge our indebtedness as a profession, and give expression to our feelings of veneration and esteem: therefore, be it

*Resolved*, That we deeply sympathize with his bereaved and sorrowing family; and as members of the same profession, acknowledging Dr. Harris as its head, we feel that we may with propriety offer this our sincere condolence.

*Resolved*, That inasmuch as his various works on Dental Science, and his self-sacrificing devotion to his profession are known to every legitimate practitioner thereof, it would be a work of supererogation for us to attempt to enumerate them. He has left ample evidences of his great ability, and an unselfish regard for his profession and humanity, which will, without our intervention, be gratefully remembered by all, and separately by every intelligent practitioner of dental surgery or medicine.

*Resolved,* As further evidence of our appreciation of the deceased, that the sum of ten dollars be contributed by this association to the "Harris Testimonial Fund."

*Resolved,* That a copy of these resolutions, signed by the committee, and attested by the officers of the association, be forwarded to the family of the deceased, and be also copied entire into the Minutes."

JOHN F. JOHNSTON,

J. F. CANINE,

P. W. MORRIS,

} *Committee.*

"WHEREAS, Death has assailed the circle of our association, and removed therefrom our highly esteemed professional brother, Dr. John Hood, of Greensburg, Ind., thus depriving us of the pleasure and benefit of association with a good and true man—a good and true dentist,—who was social, honorable and fraternal; and whereas, we, as an association of whom Dr. Hood was one among the first to identify—missing him much, and sincerely lamenting his early removal from a field of usefulness which his whole neighborhood attest he was cultivating with all his strength and ability: therefore,

*Resolved,* That the profession, and all those who came in contact with him, have cause to mourn his loss, not only out of feelings common to humanity, nor because of his professional excellence; but because he was a good citizen, in short, a useful, exemplary man, whose place his neighbors will find it difficult to fill.

*Resolved,* That we tender our warmest sympathies to the friends of the deceased, by instructing the Secretary to transmit to them a copy of the foregoing preamble and resolutions —and further *Resolved,* That they be copied into, and made a part of the record of the proceedings of this association."

JOHN F. JOHNSTON,

J. F. CANINE,

P. W. MORRIS,

} *Committee.*

On motion, the Secretary was instructed to draw on the Treasurer for the sum of ten dollars, and forward the same to the Treasurer of the "Harris Testimonial Fund," as contemplated in resolution.

The following officers were then elected for the ensuing year, to-wit: Dr. P. G. C. Hunt, of Indianapolis, *President;* Drs. Canine of Columbus, Moore of Lafayette, and Morris, of Greensburg, *Vice-Presidents*; Drs. Johnston, Hunt, Smith, Canine and Moore, *Delegates to National Association;* and Drs. M. N. Manlove, Morris, H. R. Hurd, Webster, and Knapp, *Contingent Delegates.*

Adjourned till 7½ o'clock.

7½ O'CLOCK, P. M.—The Auditing Committee reported the books and accounts of the Secretary and Treasurer are correct.

Several bills were presented and ordered to be paid.

The thanks of the association were voted to the officers of the Y. M. C. Association for the use of their room.

On resolution of Dr. Johnston, the *Constitution* was so *amended* as to provide for *semi-annual* instead of *annual* sessions.

And the association then adjourned, to meet in the city of Lafayette, on the second Tuesday of July, 1861, at 2 o'clock, P. M.

J. B. SMITH, *Rec. & Cor. Sec'y.*

